# Chit-Chat

**Published:** 1880-07

**Authors:** 


### Editorial Chit-Chat.

MARRIED.—We see by the morning paper that our friend, the veteran editor of the *Advertiser*, has been taking unto himself a wife. Congratu lations.

THE CAMPING NUMBER.—This might be called the camping number of the BISTOURY, as the Editor's portion of the labor was all performed in camp. Hence its woodsy flavor.

KEPT OUR PROMISE.—In the last number of the BISTOURY, we gave warning that the July Number would be two weeks late, by reason of our vacation in the woods. Here we are, on time.

THE DRUM MAJOR.—A little five year old, seeing a brass band led by the drum major in all his gorgeous habiliments, ran to her mother and enquired : "Ma, where did you say God lived ?" Upon receiving the mother's answer, she quickly replied : "O ! I thought He was out there with the soldiers."

THE ELMIRA MICROSCOPICAL SOCIETY, officered as follows: Dr. S. O. Gleason, President; Prof. D. R. Ford, Vice President; Dr. Thad S. Up de Graff, Sec, and Treasurer, hold regular monthly meetings, upon the first Saturday in every month, The society has 25 members.

LOCOMOTIVES AND CHURCHES.—There is a project on foot in this city to bring a railroad down to church street, locating a depot immediately in the rear of one of our principal church edifices. H., commenting upon the affair, remarked that, he "hoped the locomotive whistles would chord with the organ, else would they put the singers out."

AUSBURN TOWNER, our able contributor, has accepted the city editorship of the *Daily Free Press* of this city. This is no news to our citizens, they having long since recognized Mr. Towner's graceful pen in the excellent journal referred to. While the *Free Press* was a good paper before, it becomes superb with Mr. Towner's department.

A PIN CUSHION.—Our litte girls improvised a pin cushion that answers the purpose admirably. We are not sure but that it is an improvement of the more elaborate sort, found at home. They used a potato. It is a capital idea. We wll have one bristled with pins upon our desk at home, when we leave the woods.

MICROSCOPICAL.—"Come up, quickly," said our enthusiastic, gray-haired friend on the hillside, through the telephone, this morning; "I've got an *amœba proteus* under my sixth, and he has just surrounded a *navicula verides*, an *amphiplura pellucida;* and, a *pinnularia nobilis* makes his belly stick out like the centre pole in a circus tent. Come see him before he swallows my entire menagerie, for he can't hold out much longer without bursting his skin."

We could not find it in our heart to resist such an appeal, so we went.

DR. THAD S. UP DE GRAFF is the editor of the Elmira BISTOURY. In the January issue of that journal he has quite an extended article on "Coroners." We would suggest to the citizens of Elmira that they see to it that the author of Bodines is made coroner. If he is so fortunate as to secure an election, we hope that he may be able to retire from the Elmira Surgical Institute and give poor humanity a rest. —*Homœpathic Expositor*.

Just come over here, from under the shadow of that University, Doctor, and we'll show you we are not to be so easily suppressed—we will live on sugar-pills first.

THE NEW SCHOOL BUILDING.—We see our city has contracted for a new primary school building, to be constructed much upon the plan of the one described in the last number of the BISTOURY. Mr. Ingham, who has many excellent ideas of a school building, is the architect. We like the plan, except in the windows coming so near the floor. They should have started eight feet above the floor, instead of four, and extended to the ceiling. In this manner, would the light have been prevented from falling directly in the face. Much of the nearsightedness of children is caused by this fault of the light.

A NEST.—The ground sparrow builds a very cosey and pretty nest in a tuft of grass right in your pathway, and seems very unconcerned when you stumble over it. He will fly before you and chatter to you as though delighted that you came. He seems like a hospitable little chap but is very reckless in selecting a building site.

VALOROUS BUT INDISCREET.—An ant is an colporteur of no mean description. He can carry many times his own weight, but isa blamed fool in getting it there. He turns aside for nothing in his way. Have seen him back up a tree with a dead bug in his mandibles rather than go around. Never watched to see how long it took him to get down on the other side, but doubt not he accomplished it eventually.

FRITZ.—His friends will be glad to learn that he has distinguished himself this vacation. He has fallen into the creek, captured a woodchuck, and has developed the champion appetite of the party. He has won two boat races on the pond, having beaten an older boy who had a hundred yards the start. He knows the locality of every bird's nest about camp, and has placed a spear mark on every sucker in the pond. He has climbed the highest trees, mistaken a hornet's nest for a bird's nest, and distanced every hornet in reaching camp. While the other boys have had pains or aches, Fritz has never uttered a sound not attributable to perfect health and jolly good nature. He's a perfectly happy and contented camper.

EDISON.—This fellow, having stumbled upon three or four really useful discoveries, has become, by reason of them, a much over-estimated man. He is not an educated man to commence with, and, during his entire electric light experiments has not accomplished anything that had not been previously much better done. He seems to have been ignorant of the discoveries of others in this direction and has therefore blundered along over the same ground with his predecessors and accomplished—nothing.

Just now, he is the "inventor and proprietor" of "Edison's Polyform," a patent medicine, for the cure of neuralgia, &c. In his preparation he claims to have "bottled up" electricity in some mysterious manner, to be applied *ad libitum* to the suffering patient. If he knows anything about electricity, he knows better than to humbug the people with his "polyform."

HOUSEHOLD DUTIES.—It is well that the man should occasionally take charge of "running the house," give his wife a rest and learn what sort of an entertainment he can saddle himself with. We've tried it in camp. Have made the beds, planned and cooked the meals (by George's aid), ordered the supplies and kept everything in order for a family of ten. We are ready to testify that it's "no fool of a job," particularly with three or four children to look after and keep out of harm's way. It appears easy enough to manage a household, but, honest injun, we would rather earn the bread and let out the household management to the wife. She seems to enjoy it and we would not deprive her of any little pleasure of that sort, again, for the world.

THE FOURTH.—We thought we had escaped the day we celebrate with its accompanying noise, by fleeing to the mountains. Imagine our surprise then, upon being awakened early one morning, by the explosion of a "dolly varden" fire cracker, just outside our tent. This was speedily followed by others of less calibre, but quite as noisy during the entire day, and was supplemented by roman candles and other hissing sissing contrivances, until late into the night. The wood-robin took up his note upon the mountain top, a mile away. The whippor-wills rushed with affright from the woods, and the owls hooted dismally in some cavern far up the valley. Doubtless, this was their very first participation in a fourth of July celebration. The boys had not forgottem the day, but anticipated it by smuggling their fire-works with them.

A MIMIC.—As a comedian, an old hen partridge cannot be excelled. Come suddenly upon her when attending her young brood along the mountain road, and she will give you more illustrations of the terrible hobblings of the deformed than you would have imagined it possible for any creature to invent. She will appear with one leg crippled as from a compound-comminuted fracture; both wings paralyzed and utterly useless, hanging helpless by her side; she is blind in one eye; deaf in both ears, and generally dilapidated and used up. But try to lay hands on her, and it is wonderful how she manages to keep just out of reach. Follow her in her writhings and limpings for a half mile, then see her suddenly recover from all her ailments and fly in to the deep covert with a regular chuckle of satisfaction at having led you so far from her family of little ones. It is worth the ramble to follow her, if only to hear her note of delight.

A SUCKER, as he pokes listlessly about over the bottom of the creek, sucking the sand into his long snout and immediately squirting it out again, causing a dirty cloud all about him, and then moves on, rooting under stones and overturning sticks, forever looking for something that he never finds, strongly reminds us of some men who are continually doing the same thing. The counterpart of the sucker can be found lounging about our street corners. sucking a pipe and expectorating tobacco juice for his betters to walk in, and then vacates to get out of his own filth and to bespatter and nasty a new neighborhood.

---

"WHAT'S IN A NAME?"—We know of a gentleman who promptly answers this interrogative by exclaiming: "A hundred feet of sidewalk, at least!"

We have residing in our city two well-known gentlemen, who possess the same name. They are continually receiving each other's letters and now and then some laughable blunders occur, now in favor of one, then, the other; so that the "laughs on each other" were about equally divided, until the transaction which we now relate.

Both gentlemen possess considerable real estate, scattered about the city. The Banker ordered a sidewalk laid in front of a lot near which the Traveller also had possessions. The sidewalk builder drove in front of the Traveller's premises and inquired: "Is this Mr. ——'s property?" He was of course answered affirmatively, when he at once proceeded to displace the long sidewalk thereunto belonging. In a few days, the Traveller, surveying his premises, was surprised to see a new walk there, when it did not seem to be needed. He enquired of his agent about it, who disavowed any knowledge of it. He therefore concluded his new walk was ordered by the "other fellow," and prosecuted the inquiry no further. The Traveller has now enjoyed the walk and the joke for more than a year, while the Banker wonders why he should be ordered to lay a new walk so soon, having paid for a hundred feet of new walk to cover a fifty foot lot, only a year ago.

---

THE CITY.—The morning paper tells us of the busy people at home and how they are sweltering in the July heat. "Politics is running high." We read of the "Hancock Legion" and the "Garfield Volunteers," which smacks too much of military organization for simply political bodies of men. What nonsense all this display is, anyhow. What child's play for full grown men to indulge in. What use for all this political excitement and banding together of men to create a constant hubbub and confusion? Can not we vote just as understandingly and quite as effectively if we hold ourselves aloof from such organizations? What need of political speaking and political parades, in which much time and money is wasted? Certainly, our newspapers can keep us sufficiently informed of the questions of the day without resorting to these childish displays. What a blessing it would be to the county and its people if these clap-traps could be abolished. They are only intended to capture the weak-minded or ignorant voter. Sensible men should have nothing to do with them. Discontinue your contributions of money and keep away from them, sensible men, and they will die a natural death.

---

A FEAT IN BALANCING.—This morning we attempted to cross a rapid in the creek, over a log that reached from bank to bank. The log was moist from the dew and quite too round in form to permit of an easy or firm foot hold. We reached the centre of the impromptu bridge, however, by means of two rheumatic feet and sundry and multitudinous balancings of the arms, when a kingfisher flew just under our nose and with his unearthly chatter, startled us. We lost our centre of gravity, and, in trying to regain it, performed some very difficult feats in grotesque posturing. First, we thought we'd go in on the upper side of the log, then we changed our mind and the centre of gravity at the same time, and decided instantly upon the lower side. A unique bend in the back and a thrust of the hips backward and the shoulders forward, fashioning a capital letter Z, changed the condition of affairs again, making it extremely doubtful to ourself as well as the friend on shore [who continually volunteered all sorts of untimely remarks] which side of the blamed log would be ultimately selected for the plunge. At last, it was amicably settled between the two sides and we took an intermediate course, and sat down with considerable vehemence and most consummate skill with a leg upon *both* sides of the log. We just then observed a total eclipse of the sun and saw stars—shooting stars, all about us. This celestial phenomena lasted until we gently slid off into the babbling brook and cooled our confused brain in its crystal waters.

---

A QUIET SEAT.—While writing these items, we have sat upon a grassy bank, our feet dangling in the creek, and a fly-pole reclining upon a moss covered log, waiting for a large trout to quiet down so that we may induce him to try the fly again with which we carried away a portion of his upper lip. It is astonishing how soon they will forget an injury of that kind. No doubt, in an hour we will have him fast again, when he will probably discover what a blamed fool he was to be "taken in" in that manner again. In this, he only imitates men. We have known men to eat and drink that which many times before had nearly proved fatal to them. But, like the trout, they speedily forget the danger and pain, and try it again.

What countless living creatures one encounters when sitting thus quietly in the woods. Birds hop about in the branches over head, and look down upon you with inquisitive eyes. Kingfishers dive for minnows and newts close by your feet, throwing water over you in their plunge. Ants and many brightly colored beetles run across your paper and arrest your writing that you may scrutinize and admire them. Fish rise to take the fly that has just been bounding from point to point over the water. The golden winged woodpecker is regaleing his young with grubs that he brings momentarily from a neighboring meadow. A little speckled woodpecker is searching for insects over the trunk of a tree by your side and gives a wee grunt of satisfaction every time he impales one upon his barbed tongue. Frogs of all sizes, with voices from the deep baritone to the shrill treble, give out their single notes as though fired from a pop-gun. Butterflies take their tortuous flight through the dense thicket without ever touching a branch—a feat that would seem impossible as you look upon their course. Great brown flies alight upon your hands taxing all your fortitude to keep from spatting them that you may not frighten away the birds. There comes a muskrat, swimming gracefully up the pond. In his mouth he carries a bunch of grass, doubtless a meal for his young somewhere in the vicinity. A red squirrel comes to the brink of the water—hold still, that trout has just risen to a natural fly, under my very nose; O! ho! Wev'e got him, in the upper jaw this time, with no danger of his tearing out. How the rascal does jump and squirm and twist and shake his head. No use, old fellow, you must come to creel this time. Steady there! You can not reach that old stump—whoa, just missed it, you rascal. Well you *are* a lively chap. Want to get under that rock, hey? Let us see about that; just pull on the spring of that rod a moment. Ah, ha! That brings you to time and to the surface. Going to give up are you? Very well, just let me slip this landing net under you? No? All right, try it again, but you shall not reach that stump, unless you break rod or line. Tired are you? Well, we'll try the landing net once more. Let me reel you in a little more. There, now you are within reaching distance. What a beauty you are, and what a pity it is to take you from this charming pool. How your silver sides change to gold and ruby and emerald, and bless me, every other beautiful hue that could be mentioned. What a bright carmine your gills are, and how your pink fins harmonize with your general "make up". There, rest in the creel you handsome fellow, while we try your mate under the willow yonder; from the way he is splashing about, the chances are that he will soon occupy a position by your side.

What a picture that is before us, as we wade out into the stream! To the left, a rock bound shore overhung with long green branches of the water beech. Through them is seen the rhododendrons, their pink and white clusters peeping out everywhere. In front, a little falls, tumbling over moss-covered rocks. Beyond, a smooth sheet of water under which every stone can be distinctly seen. To the right, willows, with their yellow foliage, over which spruce and hemlock with their darker shades of green and yellow. Along the bank, an old log, completely covered with moss, and just beyond it, a cluster of "bull mint," the bright scarlet blossoms appearing all the lovelier by reason of the becoming back ground of tall, green timothy stalks that wave to and fro so gracefully in the gentle breeze. Daises, with their great round faces—so suggestive of good humor and jollity—abound everywhere. No glen seems too dark for them—no sandy plain too hot or dry—no swamp to wet. The ever cheerful daisy looks up to you from meadow, hill and dale—among the first of the flowers to salute you in the spring, the last to bid you adieu in the fall. Bless the dasies! Overhead, through the graceful branches of the trees, the clear blue sky can be seen, and the sunlight that gives constantly varying hues to the green leaves. Against a huge rock, in the foreground, wavy shadows fall, reflected from the rippling water. What a picture! How an artist would enjoy the scene and let me free to catch that trout?

